# Supplementary material for: The Impact of the Mediterranean Diet and Lifestyle Intervention on Lipoprotein Subclass Profiles among Metabolic Syndrome Patients: Findings of a Randomized Controlled Trial
Source: Int J Mol Sci. 2024 Jan 22;25(2):1338. doi: 10.3390/ijms25021338 (PMC10817060; doi:10.3390/ijms25021338)
Supplement: Supplementary file 1 [file ijms-25-01338-s001.zip › ijms-2782364-supplementary.pdf]

SUPPLEMENTARY MATERIAL

Table S1: Advanced lipoprotein profile characteristics at baseline by treatment allocation.

	Units	Control group N=94	Intervention group N=103	P value
Cholesterol				
sdLDL	mmol/L	0.60 (0.53, 0.67)	0.60 (0.52, 0.68)	0.963
sdLDL /LDL	%	16.5 (14.7,18.3)	18.3 (15.9,20.8)	0.229
VLDL	mg/dL	31.9 (25.6, 38.2)	31.5 (25.3, 37.8)	0.932
IDL	mg/dL	17.4 (15.1, 19.8)	15.4 (13.0, 17.8)	0.210
Triglycerides				
VLDL	mg/dL	113 (91, 134)	112 (86, 138)	0.967
IDL	mg/dL	16.8 (14.9, 18.8)	15.6 (13.7, 17.6)	0.380
LDL	mg/dL	21.9 (19.4, 24.5)	20.0 (17.1, 22.9)	0.295
HDL	mg/dL	25.9 (20.8, 31.1)	25.7 (21.9, 29.6)	0.950
Particle number				
VLDL	nmol/L	89.4 (72.1, 106.8)	87.8 (68.3, 107.3)	0.894
lVLDL	nmol/L	2.10 (1.75, 2.44)	2.16 (1.80, 2.52)	0.806
mVLDL	nmolL	7.23 (5.65, 8.80)	7.64 (5.62, 9.66)	0.737
sVLDL	nmolL	80.1 (64.2, 96.0)	78.0 (60.6, 95.3)	0.850
lVLDL/VLDL	%	2.46 (2.28, 2.64)	2.60 (2.39, 2.80)	0.291
mVLDL/VLDL	%	8.29 (7.28, 9.29)	8.64 (7.68, 9.60)	0.597
sVLDL/VLDL	%	89.3 (88.3, 90.2)	88.8 (87.8, 89.7)	0.456
LDL	nmolL	1490 (1350, 1630)	1356 (1244, 1468)	0.130
lLDL	nmolL	178 (162, 194)	166 (149, 183)	0.303
mLDL	nmolL	411 (348, 474)	359 (305, 412)	0.199
sLDL	nmolL	901 (815, 987)	831 (770, 891)	0.178
lLDL/LDL	%	12.1 (11.3, 12.9)	12.3 (11.4, 13.1)	0.763
mLDL/LDL	%	27.2 (24.8, 29.5)	26.0 (23.7, 28.3)	0.449
sLDL/LDL	%	60.7 (58.0, 63.5)	61.7 (58.9, 64.6)	0.588
HDL	µmol/L	29.9 (26.9, 32.9)	29.6 (27.9, 31.2)	0.819
lHDL	µmol/L	0.31 (028, 0.33)	0.28 (0.26, 0.30)	0.099
mHDL	µmol/L	9.55 (8.63, 10.47)	8.89 (8.39, 9.40)	0.214
sHDL	µmol/L	20.1 (17.9, 22.2)	20.4 (18.8, 21.9)	0.820
lHDL/HDL	%	1.04 (0.97, 1.11)	0.96 (0.88, 1.05)	0.159
mHDL/HDL	%	32.1 (30.5, 33.6)	30.4 (28.3, 32.4)	0.167
sHDL/HDL	%	66.9 (65.3,68.5)	68.7 (66.5, 70.8)	0.162
Non-HDL	nmolL	1550 (1411, 1688)	1414 (1300, 1527)	0.126
Particle size				
VLDL	nm	41.8 (41.6, 41.9)	41.8 (41.7, 42.0)	0.310
LDL	nm	20.8 (20.6, 20.9)	20.7 (20.6, 20.9)	0.747
HDL	nm	8.25 (8.22, 8.28)	8.22 (8.18, 8.25)	0.153

Data are means (95% confidence interval). sdLDL (small dense LDL); LDL (Low Density Lipoprotein); VLDL (Very Low Density Lipoprotein); IDL (Intermediate Density Lipoprotein); HDL (High Density Lipoprotein); mVLDL (medium VLDL); sVLDL (small VLDL); lLDL (large LDL); mLDL (medium LDL); sLDL (small LDL); lHDL (large HDL); mHDL (medium HDL); sHDL (small HDL); Non-HDL (Non-HDL).

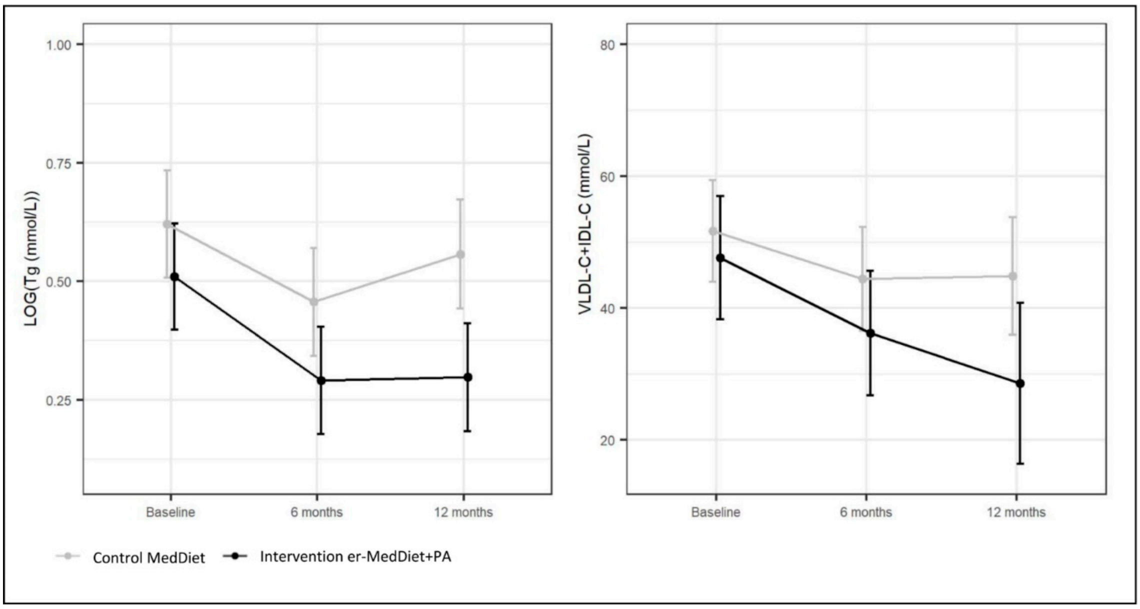

Figure S1: Effect of MetDiet and er-MetDiet+PA on Triglycerides and Remnant Lipoproteins at 6 and 12 months. LOG(Tg) (Log-transformed Triglycerides); VLDL-C (Very Low Density Lipoproteins Cholesterol); IDL-C (Intermediate Density Lipoproteins Cholesterol); MedDiet (Mediterranean Diet; er-MedDiet +PA (energy-reduced MedDiet and physical activity).

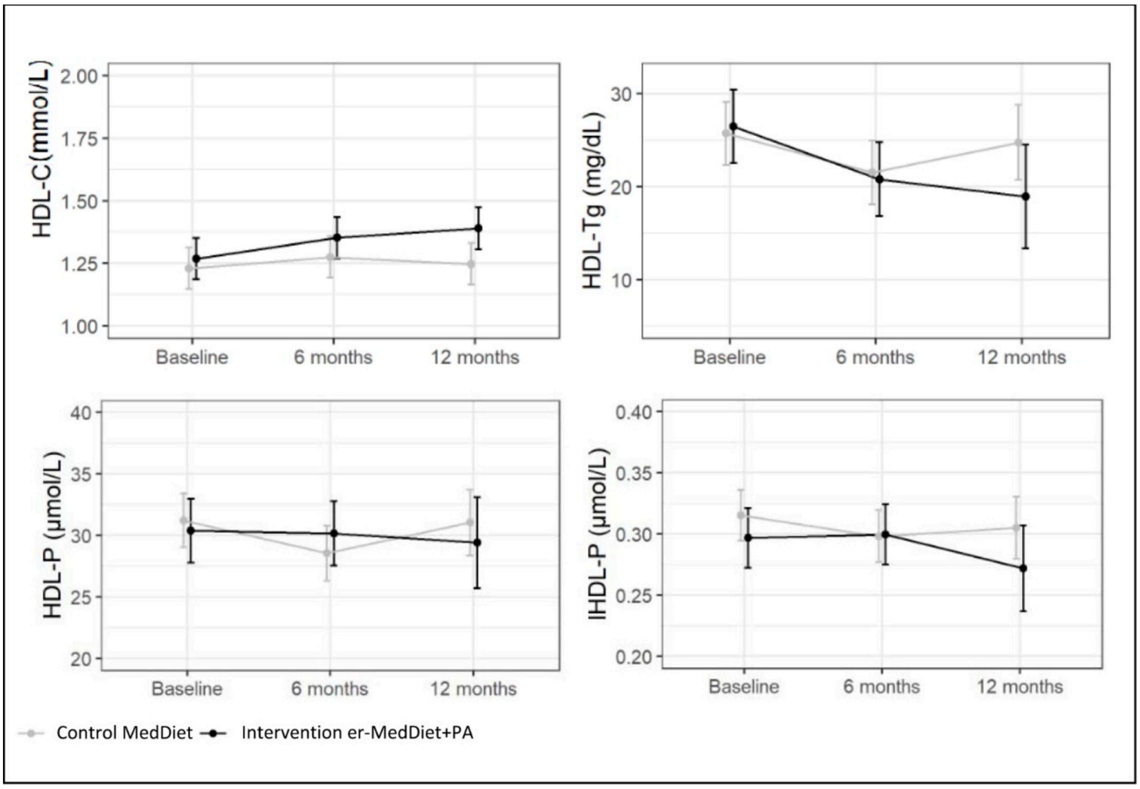

Figure S2: Effect of MetDiet and er-MetDiet+PA on HDL lipid composition and particle concentration at 6 and 12 months. HDL-C (High Density Lipoproteins Cholesterol); HDL-P (HDL Particle Number); HDL-Tg (HDL Triglycerides); lHDL-P (large HDL Particle Number); MedDiet (Mediterranean diet); er-MedDiet +PA (energy-reduced MedDiet and physical activity).

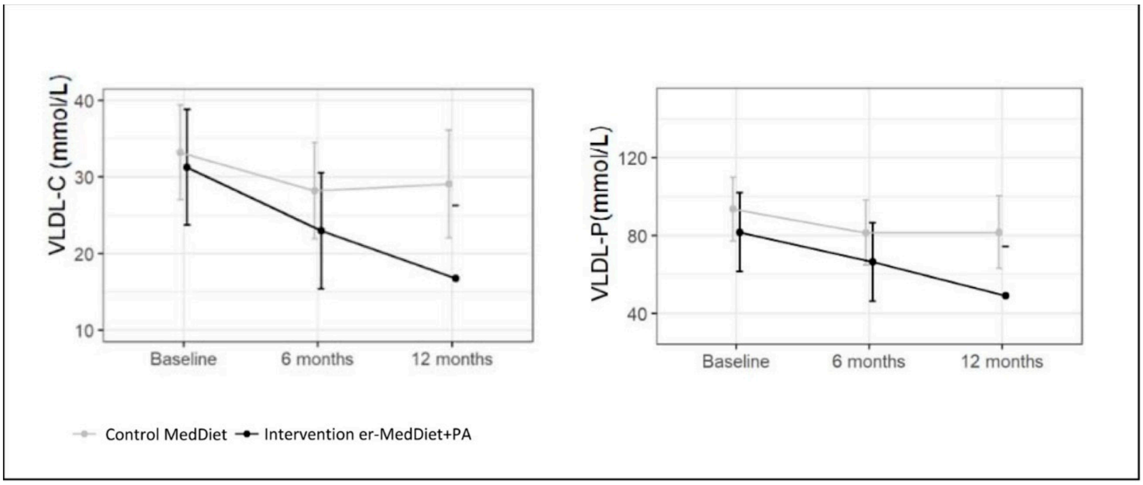

Figure S3: Effect of MetDiet and er-MetDiet+PA on VLDL advanced lipid profile results at 6 and 12 months.
VLDL-C (Very Low Density Lipoproteins Cholesterol); VLDL-P (VLDL Particle Number); MedDiet (Mediterranean diet); er-MedDiet +PA (energy-reduced MedDiet and physical activity).

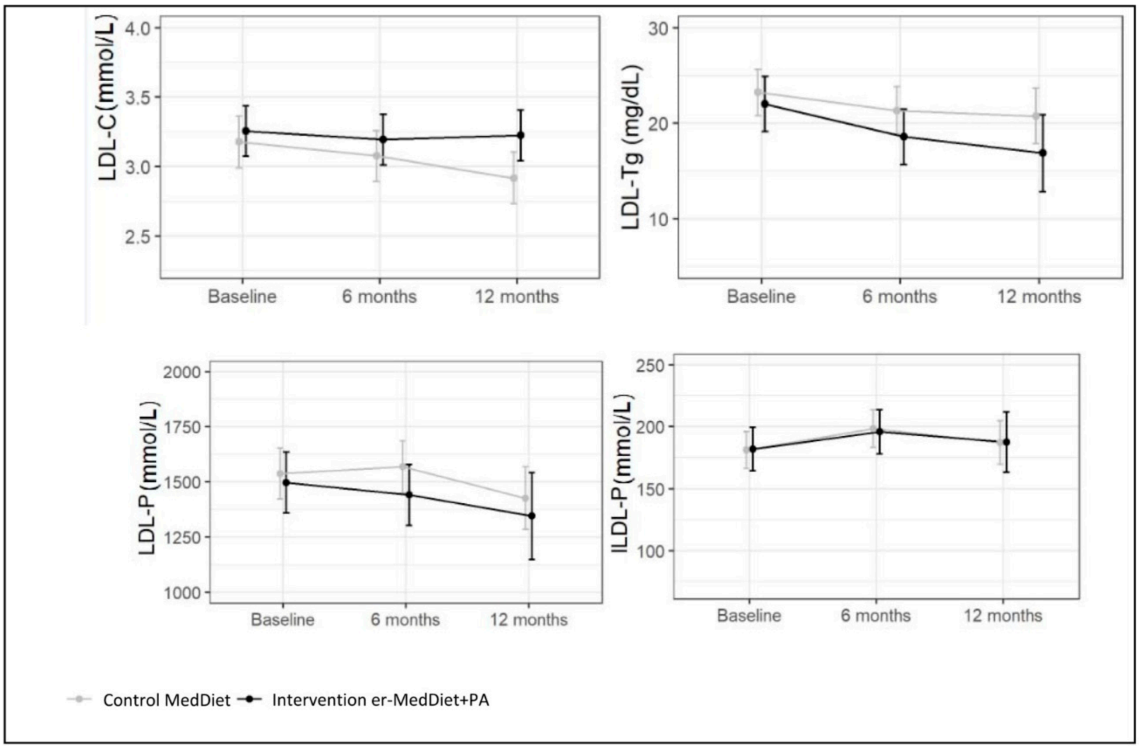

Figure S4: Effect of MetDiet and er-MetDiet+PA on LDL lipid composition and particle concentration at 6 and 12 months. LDL-C (Low Density Lipoproteins Cholesterol); LDL-P (LDL Particle Number); LDL-Tg (LDL Triglycerides); lLDL-P (large LDL Particle Number); MedDiet (Mediterranean diet); er-MedDiet +PA (energy-reduced MedDiet and physical activity).

Table S2: Effects of MedDiet and er-MedDiet+PA based intervention on lipid and anthropometric variables observed at 6 and 12 months in women.

	**Time effect** (Control effect*)				**Interaction group and time** (Intervention vs. Control effect**)			
	**6 months**		**12 months**		**6 months**		**12 months**	
	**Coefficient [95%CI]**	**P value**	**Coefficient [95%CI]**	**P value**	**Coefficient [95%CI]**	**P value**	**Coefficient [95%CI]**	**P value**
Body Mass Index (kg/m2)	-0.53[-0.92;-0.15]	**<0.01**	-0.71[-1.1;-0.33]	**<0.01**	-1.62[-2.15;-1.08]	**<0.01**	-1.41[-1.95;-0.87]	**<0.01**
Waist circumference (cm)	-2.77[-4.42;-1.12]	**<0.01**	-2.01[-3.67;-0.36]	**0.017**	-1.81[-4.11;0.5]	0.125	-2.11[-4.43;0.21]	0.074
Cholesterol								
LDL (mmol/L)	-0.06[-0.29;0.18]	0.639	-0.28[-0.52;-0.05]	**0.019**	0.1[-0.22;0.43]	0.537	0.34[0.01;0.66]	**0.045**
sdLDL (mmol/L)	-0.11[-0.29;0.07]	0.242	-0.17[-0.36;0.03]	0.089	-0.17[-0.42;0.08]	0.192	-0.03[-0.3;0.24]	0.839
HDL (mmol/L)	0.05[-0.01;0.11]	0.078	0.01[-0.04;0.07]	0.65	-0.04[-0.12;0.04]	0.346	0.07[-0.01;0.15]	0.108
Non-HDL (mmol/L)	-0.12[-0.46;0.22]	0.494	-0.54[-0.9;-0.18]	**<0.01**	0.29[-0.18;0.77]	0.228	0.8[0.27;1.32]	**<0.01**
IDL (mg/dL)	-2.59[-7.82;2.65]	0.333	-3.74[-9.34;1.85]	0.189	0.47[-6.93;7.87]	0.901	-3.22[-11.79;5.35]	0.462
VLDL (mg/dL)	-6.86[-16.86;3.15]	0.179	-8.59[-19.27;2.08]	0.115	3.05[-11.07;17.18]	0.672	-8.86[-25.22;7.51]	0.289
VLDL+IDL (mg/dL)	-9.44[-23.16;4.28]	0.177	-12.34[-26.98;2.31]	0.099	3.52[-15.85;22.9]	0.722	-12.07[-34.52;10.37]	0.292
Triglycerides								
LOG(Tg (mmol/L))	-0.12[-0.24;0]	**0.049**	-0.06[-0.17;0.06]	0.357	0.01[-0.15;0.18]	0.875	-0.06[-0.22;0.11]	0.49
LDL (mg/dL)	-1.4[-7.03;4.23]	0.626	-3.25[-9.25;2.76]	0.289	-1.2[-9.15;6.74]	0.767	-4.25[-13.45;4.96]	0.366
HDL (mg/dL)	-7.36[-15.74;1.02]	0.085	-1.2[-10.17;7.76]	0.793	2.41[-9.43;14.25]	0.69	-7.27[-21.05;6.52]	0.301
IDL (mg/dL)	-2.03[-6.11;2.04]	0.329	-2.2[-6.56;2.15]	0.321	0.44[-5.32;6.19]	0.882	-4[-10.66;2.67]	0.24
VLDL (mg/dL)	-24.59[-56.15;6.96]	0.127	-27.26[-61.27;6.75]	0.116	25.38[-19.22;69.97]	0.265	-14.64[-67.44;38.16]	0.587
Particle number								
LDL (nmol/L)	-36.98[-327.12;253.16]	0.803	-247.26[-556.99;62.47]	0.118	45.04[-364.73;454.8]	0.829	37.41[-437.26;512.08]	0.877
lLDL (µmol/L)	19.5[-13.81;52.8]	0.251	-5.54[-41.1;30.01]	0.76	-4.7[-51.73;42.34]	0.845	2.51[-51.98;57]	0.928
sLDL (nmol/L)	-65.28[-211.74;81.17]	0.382	-175.43[-334.41;-16.44]	**0.031**	74.91[-132.19;282.02]	0.478	78.19[-171.01;327.4]	0.539
HDL (µmol/L)	-5.65[-11.72;0.42]	0.068	0.02[-6.46;6.5]	0.995	4.17[-4.4;12.74]	0.34	-1.59[-11.52;8.34]	0.754
lHDL (µmol/L)	-0.03[-0.08;0.02]	0.252	-0.01[-0.07;0.04]	0.616	0.02[-0.05;0.09]	0.535	-0.02[-0.1;0.06]	0.658
sHDL (µmol/L)	-3.98[-8.26;0.3]	0.068	0.25[-4.32;4.82]	0.915	2.46[-3.58;8.5]	0.425	-2.43[-9.43;4.57]	0.496
VLDL (nmol/L)	-21.53[-48.06;5]	0.112	-24.24[-52.8;4.32]	0.096	19.76[-17.73;57.25]	0.302	-13.84[-58.1;30.41]	0.54
lVLDL (nmol/L)	-0.31[-0.87;0.25]	0.272	-0.28[-0.88;0.32]	0.365	0.28[-0.5;1.07]	0.48	-0.49[-1.42;0.44]	0.302
sVLDL (nmol/L)	-20.74[-45.3;3.82]	0.098	-22.83[-49.31;3.65]	0.091	19.36[-15.35;54.08]	0.274	-11.89[-53;29.22]	0.571
Particle size								
VLDL (nm)	0.22[-0.06;0.5]	0.13	0.27[-0.04;0.58]	0.083	-0.18[-0.58;0.22]	0.376	0.16[-0.32;0.63]	0.518
LDL (nm)	0.18[-0.08;0.44]	0.164	0.3[0.02;0.58]	**0.036**	-0.11[-0.47;0.26]	0.565	-0.11[-0.55;0.32]	0.609
HDL (nm)	0.01[-0.03;0.04]	0.791	0[-0.05;0.04]	0.876	0.02[-0.03;0.08]	0.386	0.05[-0.01;0.12]	0.109

LDL (Low Density Lipoprotein); sdLDL (small dense LDL); HDL (High Density Lipoprotein); Non-HDL (Non-High-Density Lipoprotein); IDL (Intermediate Density Lipoprotein); VLDL (Very Low-Density Lipoprotein); VLDL+IDL (Remnant Lipoproteins); Tg (Triglycerides); lLDL (large LDL); sLDL (small LDL); lHDL (large HDL); sHDL (small HDL); lVLDL (large VLDL); sVLDL (small VLDL); [IC95%] (95% confidence interval). Significant values are indicated with bold lettering. Data were analyzed by linear mixed models with intervention group, time (baseline, 6 and 12 months), interaction of group and time, and adjusted by age, the administration of lipid-lowering treatments, and smoking status. * Time effect: can be interpreted as the effect observed in the Control group. ** Interaction group and time: can be interpreted as the additional effect of the intervention group compared to the control group.

Table S3: Effects of MedDiet and er-MedDiet+PA based intervention on lipid and anthropometric variables observed at 6 and 12 months in men.

	**Time effect** (Control effect*)				**Interaction group and time** (Intervention vs. Control effect**)			
	**6 months**		**12 months**		**6 months**		**12 months**	
	**Coefficient [95%CI]**	**P value**	**Coefficient [95%CI]**	**P value**	**Coefficient [95%CI]**	**P value**	**Coefficient [95%CI]**	**P value**
Body Mass Index (kg/m2)	-0.63[-0.98;-0.27]	**<0.01**	-0.52[-0.88;-0.16]	**<0.01**	-1.3[-1.8;-0.8]	**<0.01**	-1.43[-1.94;-0.92]	**<0.01**
Waist circumference (cm)	-1.2[-2.48;0.08]	0.067	-2.43[-3.75;-1.11]	**<0.01**	-4.02[-5.83;-2.21]	**<0.01**	-2.18[-4.02;-0.33]	**0.021**
Cholesterol								
LDL (mmol/L)	-0.14[-0.36;0.07]	0.186	-0.24[-0.46;-0.01]	**0.037**	-0.03[-0.33;0.27]	0.838	0.12[-0.18;0.42]	0.447
sdLDL (mmol/L)	-0.1[-0.26;0.06]	0.23	-0.09[-0.27;0.08]	0.305	-0.25[-0.47;-0.02]	**0.03**	-0.27[-0.52;-0.02]	**0.031**
HDL (mmol/L)	0.04[-0.03;0.1]	0.246	0.02[-0.05;0.09]	0.537	0.11[0.02;0.2]	**0.019**	0.14[0.04;0.23]	**<0.01**
Non-HDL (mmol/L)	-0.08[-0.39;0.23]	0.597	-0.21[-0.56;0.13]	0.229	-0.14[-0.58;0.3]	0.531	0.32[-0.19;0.83]	0.22
IDL (mg/dL)	-2.04[-4.27;0.2]	0.074	-1.97[-4.61;0.67]	0.145	-1.79[-5.2;1.61]	0.302	-0.85[-5.49;3.8]	0.721
VLDL (mg/dL)	-4.03[-10.65;2.6]	0.234	-1.65[-9.5;6.19]	0.679	-7.3[-17.42;2.82]	0.158	-13.18[-27.02;0.66]	0.062
VLDL+IDL (mg/dL)	-6.06[-14.47;2.34]	0.157	-3.64[-13.59;6.31]	0.474	-9.16[-22;3.67]	0.162	-14.02[-31.55;3.51]	0.117
Triglycerides								
LOG(Tg (mmol/L))	-0.21[-0.34;-0.08]	**<0.01**	-0.07[-0.21;0.07]	0.321	-0.12[-0.31;0.06]	0.198	-0.24[-0.43;-0.05]	**0.014**
LDL (mg/dL)	-1.99[-4.95;0.97]	0.187	-1.87[-5.35;1.6]	0.291	-2.18[-6.68;2.31]	0.34	-1.92[-8;4.17]	0.537
HDL (mg/dL)	-2.26[-6.57;2.04]	0.302	-0.66[-5.58;4.26]	0.792	-3.77[-10.15;2.62]	0.247	-6.02[-14.46;2.43]	0.163
IDL (mg/dL)	-1.61[-3.48;0.25]	0.09	-1.39[-3.6;0.81]	0.216	-1.55[-4.4;1.29]	0.284	-0.98[-4.87;2.9]	0.62
VLDL (mg/dL)	-9.05[-36.42;18.33]	0.517	-6.95[-39.29;25.38]	0.673	-35.85[-77.58;5.87]	0.092	-66.91[-123.79;-10.02]	**0.021**
Particle number								
LDL (nmol/L)	70.12[-89.84;230.08]	0.39	-39.2[-226.51;148.11]	0.682	-171.03[-413.41;71.34]	0.167	-86.08[-413.05;240.9]	0.606
lLDL (µmol/L)	17.25[-0.35;34.85]	0.055	11.03[-9.76;31.83]	0.298	-2.65[-29.48;24.18]	0.847	2.05[-34.53;38.63]	0.913
sLDL (nmol/L)	50.44[-67.52;168.4]	0.402	-24.94[-160.4;110.52]	0.718	-160.8[-337.92;16.32]	0.075	-104.49[-337.11;128.13]	0.379
HDL (µmol/L)	-1.43[-3.8;0.94]	0.236	-0.32[-3.1;2.45]	0.819	1.64[-1.95;5.23]	0.372	0.18[-4.68;5.05]	0.941
lHDL (µmol/L)	-0.01[-0.04;0.02]	0.47	-0.01[-0.05;0.03]	0.597	0.02[-0.03;0.07]	0.428	-0.02[-0.08;0.05]	0.608
sHDL (µmol/L)	-0.8[-2.98;1.38]	0.471	0.14[-2.43;2.71]	0.914	0.36[-2.96;3.68]	0.83	0.25[-4.27;4.76]	0.914
VLDL (nmol/L)	-7.28[-25.01;10.46]	0.421	-4.76[-25.77;16.25]	0.657	-17.39[-45.21;10.44]	0.221	-28.53[-67.24;10.18]	0.149
lVLDL (nmol/L)	-0.07[-0.43;0.29]	0.706	0[-0.43;0.43]	0.99	-0.57[-1.12;-0.01]	**0.046**	-0.97[-1.73;-0.21]	**0.013**
sVLDL (nmol/L)	-5.76[-25.03;13.51]	0.558	-4.1[-26.87;18.67]	0.724	-7.66[-37.03;21.71]	0.609	-7.67[-47.73;32.38]	0.707
Particle size								
VLDL (nm)	-0.04[-0.22;0.14]	0.652	-0.01[-0.22;0.2]	0.926	0.18[-0.1;0.46]	0.212	0.27[-0.09;0.63]	0.143
LDL (nm)	0.01[-0.21;0.24]	0.901	0.05[-0.21;0.31]	0.72	0.24[-0.1;0.58]	0.171	0.15[-0.3;0.6]	0.517
HDL (nm)	0[-0.04;0.03]	0.822	-0.02[-0.06;0.02]	0.37	-0.01[-0.07;0.05]	0.641	0.02[-0.06;0.1]	0.586

LDL (Low Density Lipoprotein); sdLDL (small dense LDL); HDL (High Density Lipoprotein); Non-HDL (Non-High-Density Lipoprotein); IDL (Intermediate Density Lipoprotein); VLDL (Very Low-Density Lipoprotein); VLDL+IDL (Remnant Lipoproteins); Tg (Triglycerides); lLDL (large LDL); sLDL (small LDL); lHDL (large HDL); sHDL (small HDL); lVLDL (large VLDL); sVLDL (small VLDL); [IC95%] (95% confidence interval). Significant values are indicated with bold lettering. Data were analyzed by linear mixed models with intervention group, time (baseline, 6 and 12 months), interaction of group and time, and adjusted by age, the administration of lipid-lowering treatments, and smoking status. * Time effect: can be interpreted as the effect observed in the Control group. ** Interaction group and time: can be interpreted as the additional effect of the intervention group compared to the control group.

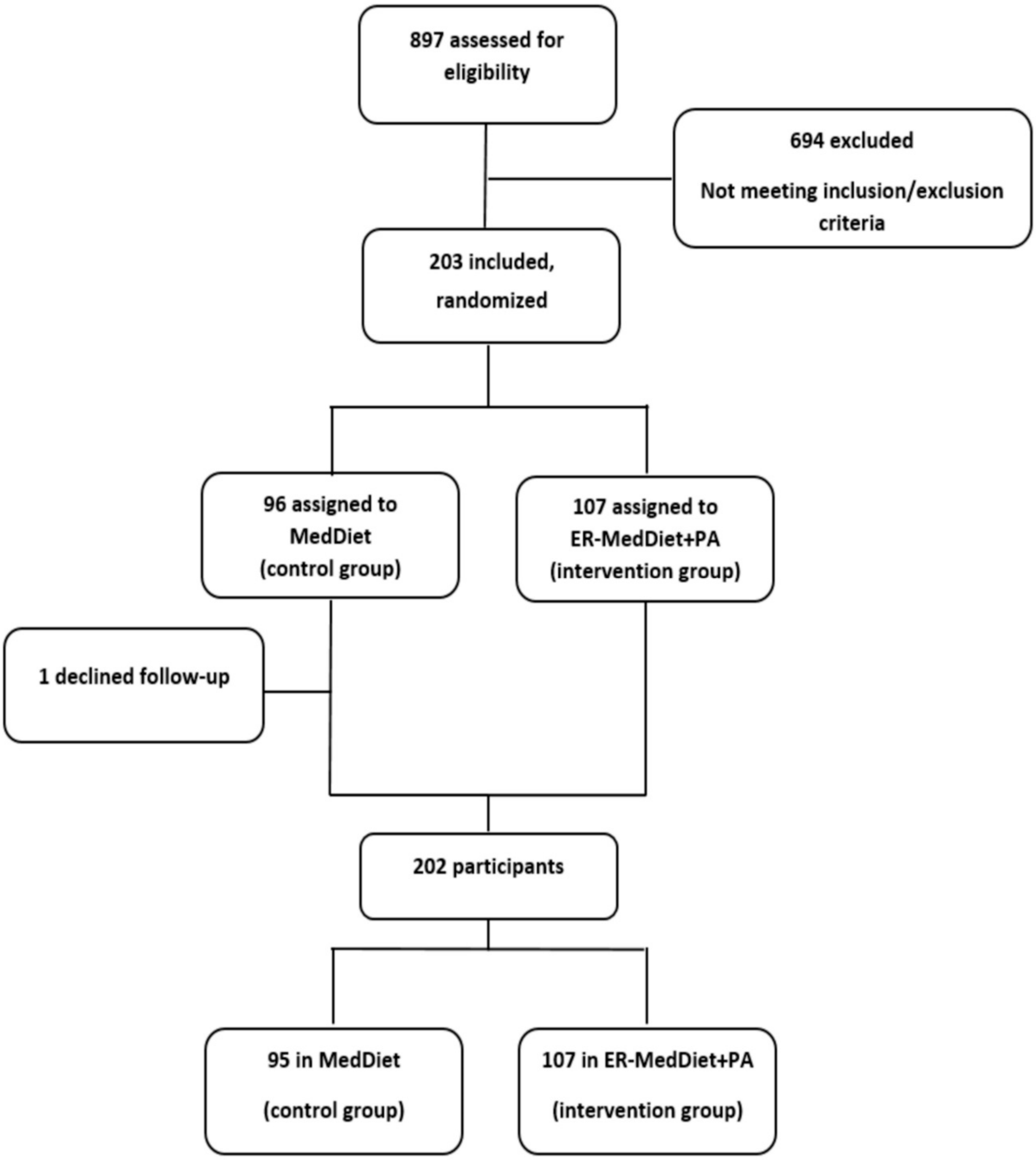

Figure S5: Trial flow chart.
MedDiet (Mediterranean diet); er-MedDiet +PA (energy-reduced MedDiet and physical activity).
